# A Multicolor Fluorescence *in situ* Hybridization Approach Using an Extended Set of Fluorophores to Visualize Microorganisms

**DOI:** 10.3389/fmicb.2019.01383

**Published:** 2019-06-19

**Authors:** Michael Lukumbuzya, Markus Schmid, Petra Pjevac, Holger Daims

**Affiliations:** ^1^Centre for Microbiology and Environmental Systems Science, Division of Microbial Ecology, University of Vienna, Vienna, Austria; ^2^The Comammox Research Platform, University of Vienna, Vienna, Austria

**Keywords:** fluorescence *in situ* hybridization, confocal laser scanning microscopy, white light laser, fluorophores, microorganisms, three-dimensional structure, activated sludge

## Abstract

Fluorescence *in situ* hybridization (FISH) with rRNA-targeted oligonucleotide probes is a key method for the detection of (uncultured) microorganisms in environmental and medical samples. A major limitation of standard FISH protocols, however, is the small number of phylogenetically distinct target organisms that can be detected simultaneously. In this study, we introduce a multicolor FISH approach that uses eight fluorophores with distinct spectral properties, which can unambiguously be distinguished by confocal laser scanning microscopy combined with white light laser technology. Hybridization of rRNA-targeted DNA oligonucleotide probes, which were mono-labeled with these fluorophores, to *Escherichia coli* cultures confirmed that the fluorophores did not affect probe melting behavior. Application of the new multicolor FISH method enabled the differentiation of seven (potentially up to eight) phylogenetically distinct microbial populations in an artificial community of mixed pure cultures (five bacteria, one archaeon, and one yeast strain) and in activated sludge from a full-scale wastewater treatment plant. In contrast to previously published multicolor FISH approaches, this method does not rely on combinatorial labeling of the same microorganisms with different fluorophores, which is prone to biases. Furthermore, images acquired by this method do not require elaborate post-processing prior to analysis. We also demonstrate that the newly developed multicolor FISH method is compatible with an improved cell fixation protocol for FISH targeting Gram-negative bacterial populations. This fixation approach uses agarose embedding during formaldehyde fixation to better preserve the three-dimensional structure of spatially complex samples such as biofilms and activated sludge flocs. The new multicolor FISH approach should be highly suitable for studying structural and functional aspects of microbial communities in virtually all types of samples that can be analyzed by conventional FISH methods.

## Introduction

Fluorescence *in situ* hybridization (FISH) targeting ribosomal RNA is a widely used molecular tool for the cultivation-independent identification, visualization, and quantification of microorganisms in environmental and medical samples. In standard FISH approaches, fluorescently mono-labeled oligonucleotide probes are hybridized to the rRNA of microbial cells, and the stained cells are subsequently visualized by widefield epifluorescence or confocal laser scanning microscopy (e.g., Amann et al., 1990; Wagner et al., 1994). A key advantage is that FISH (and methods derived from FISH) reveals the localization of the targeted microorganisms within complex environments, such as biofilms, and their colocalization patterns with other organisms (Daims et al., 2006; Almstrand et al., 2013; Mark Welch et al., 2016; Probandt et al., 2018). Spatial distribution and colocalization data help identify and characterize specific microbe-microbe interactions. Examples include syntrophic symbioses (Maixner et al., 2006), predator-prey relationships (Dolinšek et al., 2013), and interspecies cooperation during infection (Stacy et al., 2014). When combined with single-cell isotope labeling and chemical imaging techniques to study microbial physiology *in situ* (Lee et al., 1999; Wagner, 2009), FISH offers unique possibilities to relate spatial information with the metabolic capabilities of uncultured microorganisms (Berry et al., 2013).

Approaches based on FISH, which are applied to analyze the abundance, spatial distribution, and physiology of microorganisms, will benefit if many different target organisms can be visualized in the same experiment. The more organisms can be detected by FISH simultaneously, the less time and sample material is needed for sample preparation, *in situ* hybridization, fluorescence image acquisition, and if applicable, additional analyses such as chemical imaging (Wagner, 2009). However, in standard FISH applications up to three different fluorophores are used for mono-labeling up to three different oligonucleotide probes at the 5′-ends. Therefore, not more than three phylogenetically distantly related target organisms can be detected concurrently. Only if the target organisms represent different clades within the same phylogenetic group, so that probes specific for this group and sub-groups can be hierarchically nested, up to seven populations can be distinguished based on the probe binding patterns (Amann et al., 1996).

The fluorophores most commonly used for standard FISH are the sulfoindocyanine dyes Cy3 and Cy5, and 5(6)-carboxyfluorescein-N-hydroxysuccinimide ester (FLUOS). The limitation to these three dyes (or to fluorophores with similar excitation and emission spectral properties) is mainly caused by the use of band- or long-pass filters in fluorescence microscopy, and problems with excitation crosstalk and emission bleed-through of fluorophores (Valm et al., 2012). Several methods have recently been developed to overcome this limitation. The multicolor DOPE-FISH approach uses oligonucleotide probes that are double-labeled with different fluorophores at their 5′- and 3′-ends (Stoecker et al., 2010; Behnam et al., 2012). It combines the simplicity of the standard FISH protocol with new label combinations to visualize up to six phylogenetically distantly related microbial taxa in the same experiment. A conceptually similar method relies on quadruple-labeled oligonucleotide probes (Schimak et al., 2015). Here, up to four fluorophores are attached to multi-labeled (MiL)-FISH probes by CLICK chemistry. The fluorescence signal intensities of the tagged microorganisms are comparable or higher than intensities achieved by standard FISH with mono-labeled probes.

Double- or quadruple-labeling of the same microbial cell with different dyes leads to color blending, and the blended colors are used to identify the target organisms (we refer to this approach as “combinatorial labeling”). However, a similar signal intensity (brightness) of all fluorophores in the recorded images is an important prerequisite for correct color blending in multicolor DOPE-FISH or MiL-FISH experiments. In order to meet this condition, the imaging parameters (e.g., camera exposure time or fluorescence detector sensitivity) must be carefully adjusted for each dye. This can be difficult if target populations, which are labeled by different dye combinations that include one identical fluorophore, strongly differ in their fluorescence brightness. For example, different signal intensities can be caused by a different cellular ribosome content, a different efficiency of probe binding to their target sites, and a different cell wall permeability for oligonucleotide probes (Wagner et al., 2003).

CLASI-FISH is an approach to achieve the simultaneous detection of numerous different taxa, which uses combinatorial labeling with different dyes and spectral imaging (Valm et al., 2012; Mark Welch et al., 2016). A unique combination of two or more distinctly mono-labeled probes is used to tag each single microbial taxon, so that the taxa are distinguished from each other by the spectral properties of the combined fluorophores. The taxa are then identified by applying linear unmixing during post-processing of the spectral images (Valm et al., 2012). CLASI-FISH has been successfully applied to distinguish up to 15 different target organisms at once (Valm et al., 2011; Mark Welch et al., 2016). However, the application of two distinct oligonucleotide probes to label the same microorganism in CLASI-FISH experiments, requires the binding affinities of the applied probes to be highly similar. If this requirement is not met, the sensitivity of the assay might decrease due to shifts in the spectral composition of the fluorescent signals (Behnam et al., 2012).

For reasons outlined above, all currently available multicolor FISH methods still use a small set of different fluorophores and then rely on combinatorial labeling, which can be challenging to interpret correctly. Problems with recording the fluorescence of specific fluorophores can occur if samples show strong autofluorescence at the respective wavelengths (Behnam et al., 2012), drastically reducing the number of targets detectable by any of the above described methods. However, none of these methods exploit the full potential of state-of-the-art fluorescence microscopy. Confocal laser scanning microscopes (CLSMs) with white light laser (WLL) technology, which provide seamlessly tunable excitation wavelengths across almost the entire UV-VIS spectrum (470 to 670 nm) and freely definable windows for the recorded emission wavelengths are a recent development in the field of epifluorescence microscopy. At the same time, highly sensitive hybrid photodetectors, which allow the acquisition of fluorescence signals even when very narrow emission wavelength windows are defined to reduce bleed-through, are now available (Borlinghaus et al., 2012). These features enhance the flexibility of both fluorophore excitation and emitted fluorescence detection, and thus they extend the range of fluorophores one can utilize for FISH and other fluorescence imaging approaches.

Here we introduce a multicolor FISH approach, which utilizes the flexibility of WLL technology to detect multiple microbial populations without combinatorial labeling. The method is based on the standard FISH protocol with mono-labeled oligonucleotide probes but uses an extended set of fluorophores with distinct spectral properties spanning almost the entire spectrum of visible light. We demonstrate the successful application of this multicolor FISH approach to an artificial mixture of cultured microorganisms (referred to as “mock community”) and to a complex microbial community in activated sludge from a municipal wastewater treatment plant (WWTP). All described experiments were performed using a CLSM equipped with a WLL and a UV diode. The novel multicolor FISH method can in principle also be used with more commonly available CLSMs (equipped with Ar and HeNe lasers and a UV diode) and with conventional epifluorescence microscopes. However, the number of simultaneously applicable fluorochromes depends on the microscope setup (e.g., number of installed lasers or filter sets) and is most likely smaller than with a WLL.

## Materials and Methods

### Cultivation of Reference Organisms, Activated Sludge Sampling, and Cell Fixation

*Escherichia coli* K12 (DSM 498) and *Bacillus subtilis* WS29 (DSM 10) were grown at 37°C in lysogeny broth. *Niabella soli* (DSM 19437) was grown at 37°C in R2A medium (DSMZ medium 830). The nitrite-oxidizing bacterium “*Candidatus* Nitrotoga fabula” was cultured in nitrite-containing mineral medium as detailed elsewhere (Kitzinger et al., 2018). The complete ammonia oxidizer (comammox organism) *Nitrospira inopinata* (Daims et al., 2015) and the ammonia-oxidizing archaeon *Nitrososphaera gargensis* were grown in mineral ammonium-containing media (Palatinszky et al., 2015; Kits et al., 2017). *Saccharomyces cerevisiae* DSM 70449 was grown at 37°C in DSMZ medium 393. The four Gram-negative bacteria *E. coli*, *N. soli*, “*Candidatus* N. fabula,” and *N. inopinata*, and the yeast *S. cerevisiae* were harvested during the late logarithmic growth phase by centrifugation, washed once in phosphate-buffered saline (1 × PBS), and fixed in a 2% (v/v) formaldehyde solution for 4 h at 4°C as described elsewhere (Daims et al., 2005). *B. subtilis* and *N. gargensis* cells were harvested during the late logarithmic growth phase by centrifugation and fixed in a 1:1 mixture of 1 × PBS and 96% (v/v) ethanol according to Daims et al. (2005).

Activated sludge was sampled in March 2015 from a nitrifying-denitrifying tank at the municipal WWTP of Klosterneuburg, Austria. The sludge was centrifuged (20,817 ×*g*, 4°C, 15 min), most of the supernatant was removed, and the samples were fixed in a 2% (v/v) formaldehyde solution for 3 h at 4°C. Fixed samples were washed in 1 × PBS, resuspended in a 1:1 mixture of 1 × PBS and 96% (v/v) ethanol (Daims et al., 2005), and stored at -20°C. In addition, a modified fixation protocol was tested to better preserve the spatial structure of activated sludge flocs. For this purpose, sludge was sampled from the same WWTP in September 2016 and was suspended on site in a 1:1 mixture of 0.1% (w/v) LE agarose [Biozyme, gelling strength (1%) ≥ 1,200 g/cm^2^] and 2% (v/v) formaldehyde solution. The mixture was incubated for 1 h at ambient temperature. After transfer to the laboratory, the sludge suspended in agarose was transferred onto microscope slides and air-dried at room temperature for approximately 30 min (the agarose solidified during this step). To remove the fixative, the slides were dipped twice into 1 × PBS for 10 min each. Subsequently, the slides were dipped for 5 min each into 50, 70, and 96% (v/v) ethanol for dehydration and were stored at -20°C.

To test the applicability of the fluorophores in a sequential hybridization setup, an activated sludge sample with a previously described high diversity of *Nitrospira*-related nitrite-oxidizing bacteria (NOB) was used (Gruber-Dorninger et al., 2015). This nitrifying activated sludge had been sampled from a sequencing batch reactor, which treats reject water from sludge dewatering after anaerobic digestion, at the municipal full-scale WWTP of Ingolstadt (Germany) in October 2009, had been fixed by the same conventional protocol as described above (Daims et al., 2005), and had been stored at -20°C.

### Oligonucleotide Probes and Fluorophores

The rRNA-targeted DNA oligonucleotide probes used in this study are listed in Table 1. The new probe Nia224 (targeting the genus *Niabella*) was designed using the “probe design” and “probe match” functions of the software ARB (Ludwig et al., 2004) and the SILVA NR99, release 128 16S rRNA sequence database (Quast et al., 2013). Probes were mono-labeled at their 5′-ends with fluorophores as listed in Table 1. Molecular and spectral properties of all fluorophores used in this study are provided in Table 2. Fluorescently labeled probes and unlabeled competitor oligonucleotides (Table 1) were obtained from Biomers (Ulm, Germany).

**Table 1:** Ribosomal RNA-targeted oligonucleotide probes used in this study.

Probe	Fluorophore^a^	Target organisms	Probe sequence (5′-3′)	Competitor sequence (5′-3′)^b^	FA^c^ (%)	References
L-C-gProt-1027-a-A-17 (Gam42a)	Atto 490 LS, Atto 633	Gammproteobacteria	GCCTTCCCAC ATCGTTT	GCCTTCCCAC TTCGTTT	35	Manz et al., 1992
LGC354A	Atto 633, FLUOS	Firmicutes	TGGAAGATTCC CTACTGC		35	Meier et al., 1999
LGC354B	Atto 633, FLUOS	Firmicutes	CGGAAGATTC CCTACTGC		35	Meier et al., 1999
LGC354C	Atto 633, FLUOS	Firmicutes	CCGAAGATTCC CTACTGC		35	Meier et al., 1999
EUK1195	Atto 565	Eukaryotes	GGGCATCACAGACCTG		35	Giovannoni et al., 1988
S-D-Bact-0338-a-A-18 (EUB338-I)	all dyes	most Bacteria	GCTGCCTCCC GTAGGAGT		35	Amann et al., 1990
S-^∗^-BactP-0338-a-A-18 (EUB338-II)	all dyes	Planctomycetales	GCAGCCACCC GTAGGTGT		35	Daims et al., 1999
S-^∗^-BactV-0338-a-A-18 (EUB338-III)	all dyes	Verrucomicrobia	GCTGCCACCC GTAGGTGT		35	Daims et al., 1999
NONEUB	all dyes	control probe	ACTCCTACGGGA GGCAGC		35	Wallner et al., 1993
S-D-Arch-0915-a-A-20 (Arch915)	DY-681	Archaea	GTGCTCCCCCGCC AATTCCT		35	Amann et al., 1990
Ntoga221	Atto 594	genus *Nitrotoga*	TATCGGCCGCTC CGAAAA	CATCGGCCGCTCC GAAAG	35	Lücker et al., 2015
S-^∗^-Ntspa-1431-a-A-18 (Ntspa1431)	Atto 532	*Nitrospira* lineage I	TTGGCTTGGGCG ACTTCA		35	Maixner et al., 2006
S-G-Ntspa-662-a-A-18 (Ntspa662)	Cy3, FLUOS	genus *Nitrospira*	GGAATTCCGCGCT CCTCT	GGAATTCCGCTC TCCTCT	35	Daims et al., 2001
S-^∗^-Ntspa-712-a-A-21 (Ntspa712)	Cy3, FLUOS	phylum *Nitrospirae*	CGCCTTCGCCACC GGCCTTCC	CGCCTTCGCCACCG GTGTTCC	35	Daims et al., 2001
Ntspa1131	Atto 594	*Nitrospira* Cluster Ib	GTGCTCGGCTT GACCCGG	GTGCTCGGCATGA CCCGG	50	Gruber-Dorninger et al., 2015
Ntspa451	Atto 633	*Nitsospira* Cluster Ig	AGCAGTTACCTGC CCCAT		25	Gruber-Dorninger et al., 2015
Ncom1025	Atto 490LS, FLUOS	*Nitrosomona communis* lineage	CTCGATTCCCT TTCGGGCA		35	Juretschko, 2000
Cl6a192	DY-681, FLUOS	*Nitrosomonas oligotropha* lineage	CTTTCGATCCCC TACTTTCC	CTTTCGATCCCCTGC TTTCC	35	Adamczyk et al., 2003
S-F-bAOB-1224-a-A-20 (Nso1225)	FLUOS	betaproteobacterial ammonia-oxidizing bacteria	CGCCATT GTATTACGTGTGA		35	Mobarry et al., 1996
S-^∗^-Nsm-0651-a-A-18 (NEU)	FLUOS	most halophilic and halotolerant *Nitrosomonas* spp.	CCCCTCTGCT GCACTCTA	TTCCATCCCCC TCTGCCG	40	Wagner et al., 1994
CF193a	Atto 594	*Flavobacteria*, *Bacteroidetes*, *Sphingobacteria*	TGGTCCGTGTCTC AGTAC		35	Manz et al., 1996
S-G-Nia-224-a-A-18 Nia224	Atto 532	genus *Niabella*	ATACGCACACCC GTCTTC		35	This study

*^a^Fluorophore(s) used for labeling the respective probes. ^b^Competitors were added as unlabeled oligonucleotides and in equimolar concentrations as the labeled probes. ^c^FA, formamide. The formamide concentration in the hybridization buffer (v/v) is indicated*.

**Table 2:** Spectral and molecular properties of the fluorophores used for labeling rRNA-targeted oligonucleotide probes.

Fluorophore	λ_max_ (excitation)	λ_max_ (emission)	Detection window (nm)^a^	MW^b^ (g/mol)
Atto 425^c^	439 nm	485 nm	475–495	401
Atto 490LS	496 nm	661 nm	645–685	696
Atto 532	532 nm	552 nm	545–565	765
Atto 565	564 nm	590 nm	580–600	611
Atto 594	603 nm	626 nm	620–640	1137
Atto 633	630 nm	651 nm	650–670	652
DY-681^d^	691 nm	708 nm	700–720	737
FLUOS	492 nm	517 nm	510–530	473
Cy3	554 nm	668 nm	550–570	767
Cy5	649 nm	666 nm	660–670	754

*^a^Wavelength windows used for recording the emitted fluorescence. ^b^MW, molecular weight. ^c^For Atto 425, an excitation wavelength of 405 nm was used. ^d^For DY-681, an excitation wavelength of 670 nm was used*.

### FISH and Microscopy

FISH of pure cultures of *E. coli*, of mixed pure cultures of reference organisms (mock community), and of all activated sludge samples was performed at 46°C according to a standard protocol for rRNA-targeted FISH (Manz et al., 1992; Daims et al., 2005) with the following modifications. Prior to application of the hybridization buffer onto the microscope slide, the buffer was mixed with a 5 to 15 pmol/μl working stock of each applied probe (Table 1) at a buffer: probe ratio of 10:1 (v/v). The hybridizations were performed for 2 h for pure cultures and the mock community samples, 2 h for each of three sequential hybridizations on sludge samples from the WWTP Ingolstadt or 12 h for single hybridizations on activated sludge samples from the WWTP Klosterneuburg. The long hybridization time of 12 h was chosen to improve the signal intensity of some of the applied probes (Yilmaz et al., 2006).

Following FISH, images were acquired with an inverted Leica TCS SP8X CLSM equipped with a 405 nm UV diode, a Leica supercontinuum white light laser, two photomultiplier (PMT) detectors, three hybrid (HyD) detectors, and the Leica Application Suite AF 3.2.1.9702. Fluorophores were excited at the respective excitation wavelength (Table 2) with the fixed wavelength diode laser or the WLL, respectively. The wavelength windows for recording the emitted fluorescence were adjusted according to the light emission properties of the fluorophores (Table 2).

The separately recorded fluorescence signals of the fluorophores were false-colored and overlaid to form a composite image by using the Leica Application Suite X and the image analysis and visualization software *daime* version 2.1 (Daims et al., 2006). To visualize multicolor FISH of activated sludge, three-dimensional (3D) confocal image stacks (*z*-stacks) of each fluorophore signal were recorded at the same position in the sample. The *z*-stacks were then false-colored and visualized together by maximum intensity projection (MIP) rendering in *daime*. MIP would accumulate the autofluorescence background of the sludge matrix from all images of all stacks in the final projection. To mitigate this effect prior to visualization, the images of the different fluorophore signals were subtracted from each other using the respective tool of the *daime* program. Image subtraction ensured that fluorescent background, which occurred in two or more images at the same position, was reduced while the specific probe signals were qualitatively preserved.

### Evaluation of Fluorophore Intensity, Excitation Crosstalk, and Emission Bleed-Through

To compare the fluorescence intensities of the different fluorophores after rRNA-targeted FISH, aliquots of fixed *E. coli* cells from the same culture were separately hybridized to probe EUB338-I labeled with the different fluorophores (Table 2). The formamide concentration in the hybridization buffer was set to 35% in these experiments, and all hybridizations were performed in parallel. For each fluorophore, images of probe-stained cells were recorded using the respective settings for excitation and emission wavelengths. Other parameters of image acquisition were kept constant (in particular, the laser power was set to 10% and the “smart gain” was set to 100%). The mean fluorescence intensities of probe-stained cells were measured by image analysis using *daime*. To evaluate excitation crosstalk and emission bleed-through for the dyes Cy3, Atto 532, and Atto 565 as well as for Cy5 and Atto 633, *E. coli* cells were again separately hybridized to probe EUB338-I labeled with each of these dyes. Subsequently, images of probe-conferred fluorescence were acquired using the excitation wavelengths and emission recording windows of every dye (e.g., cells labeled with Cy3 were imaged with the settings for Cy3, Atto 532, and Atto 565). The mean fluorescence intensities of probe-stained cells were then measured by using *daime*. All data were plotted using R (R Core Team, 2017).

### Dissociation Profiles of Oligonucleotide Probes

Fixed *E. coli* cells were separately hybridized to probe EUB338-I labeled with Cy3, Cy5, FLUOS, and the alternative fluorophores (Table 2) with increasing formamide concentrations in the hybridization buffer [0 to 70% (v/v) with 10% increments] as described elsewhere (Manz et al., 1992; Daims et al., 2005). Images of the fluorescent cells were recorded, and the probe dissociation profiles were determined by image analysis using the respective function of the *daime* program. The measured data were plotted and the probe dissociation curves were approximated by non-linear regression with a sigmoidal regression model in R. To determine the optimal hybridization conditions for the new probe Nia224 (Table 1), the same approach was used with fixed cells of *Niabella soli* and probe Nia224 labeled with Cy3.

### Spatial Arrangement Analysis of Nitrifiers in Activated Sludge

Activated sludge samples from the WWTP Klosterneuburg were formaldehyde-fixed by the standard fixation protocol or by using a modified protocol with agarose embedding (see above). The sludge samples were hybridized to a mixture of probes Nso1225, Ncom1025, Cl6a192, and NEU (all labeled with FLUOS) to detect betaproteobacterial ammonia-oxidizing bacteria (AOB) and simultaneously to a mixture of probes Ntspa662 and Ntspa712 (both labeled with Cy3) to detect NOB of the genus *Nitrospira* (see also Table 1). Following FISH, confocal *z*-stacks of probe-conferred fluorescence were acquired at randomly chosen positions within each specimen. Twenty-three *z*-stacks were recorded for conventionally fixed and for agarose-embedded sludge, respectively. Each *z*-stack comprised the whole vertical thickness of the sludge floc at the respective position. This vertical thickness was determined manually, by finding the *z*-positions where the biomass at the top and the bottom of a floc was just outside the focal plane of the microscope. The *xy* resolution of the images was 1024 × 1024 pixels, and the axial distance between the images in the *z*-stacks was 0.34 μm. The spatial arrangement patterns of AOB and NOB in the *z*-stacks were analyzed by using the 3D “Inflate algorithm” of the *daime* software. Briefly, this approach quantifies the density of one microbial population (here: “population A”) at increasing distances from the cells (or cell clusters) of another population (“population B”). The measured densities of population A are normalized with the expected densities of a virtual population A’, which is equally abundant in the sample but has a random spatial distribution (i.e., random distribution is the null hypothesis). A normalized density of the real population A > 1.0 indicates coaggregation of populations A and B, a density < 1.0 indicates avoidance, and a density of 1.0 indicates random distribution at the respective distance. Details of the algorithm are described elsewhere (Daims and Wagner, 2011; Dolinšek et al., 2013). Prior to analysis, the *xy* resolution of the images was reduced in *daime* to 512 × 512 pixels in order to save computation time (image data were preserved by trilinear interpolation). A 3D median filter (kernel size 3 × 3 × 3 voxels) was applied to reduce background noise and to eliminate spurious small particles from the images.

### Sequential Hybridization of *Nitrospira* in Activated Sludge

Activated sludge samples from the WWTP Ingolstadt were hybridized with three different rRNA-targeted DNA oligonucleotide probes specific to NOB of *Nitrospira* sublineage Ib (Ntspa1131), *Nitrospira* lineage I (Ntspa1431), and *Nitrospira* sublineage Ig (Ntspa451), with hybridization optima at three distinct formamide concentrations (Table 1). The first hybridization step was performed for 2 h at a formamide concentration of 50% with probe Ntspa1131 (labeled with Atto 594), followed by a 10 min washing step. Immediately thereafter, two more 2 h hybridization and 10 min wash cycles at 35% (Ntspa1431, labeled with Atto 532) and 25% (Ntspa451, labeled with Atto 633) formamide concentration were performed.

## Results

### Evaluation of Alternative Fluorophores for rRNA-Targeted FISH of Microbial Cells

**FIGURE 1:**
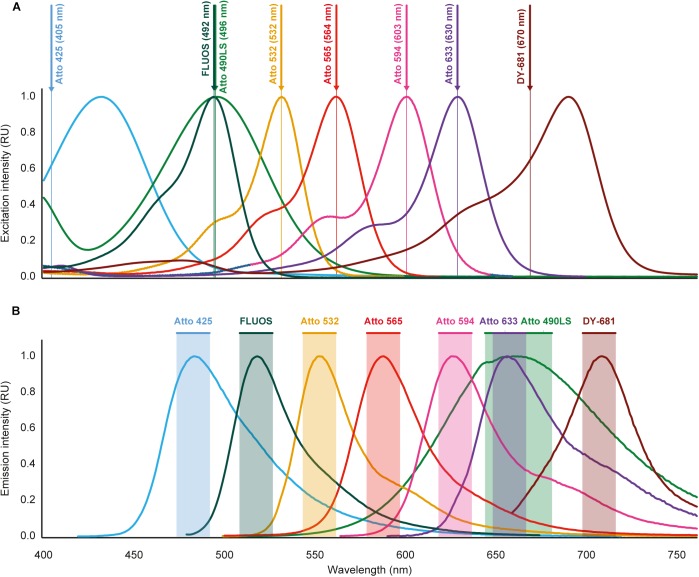
Excitation and emission spectra of the fluorochromes used for multicolor FISH in this study. **(A)** Excitation spectra. Wavelengths indicated after the fluorochrome names and by vertical lines are the excitation wavelengths applied for imaging. For Atto 425 and DY-681, different wavelengths than the maxima of the excitation spectra were used because of technical limitations of the microscopy equipment. **(B)** Emission spectra. Shaded regions indicate the wavelength windows used for recording the fluorescence emitted by the respective dyes. The spectral properties of the fluorochromes are listed in Table 2. Please refer to Fig. S1 for an illustration that includes the spectra of the widely used dyes Cy3 and Cy5, which were not used for multicolor FISH because of overlapping spectra with several of the other fluorochromes (see text for details). RU, relative units.

**FIGURE 2:**
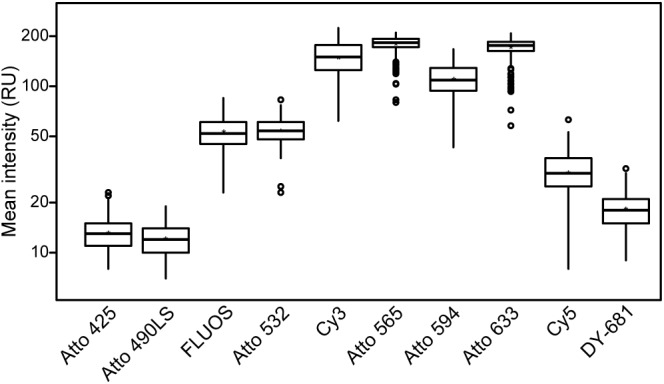
Comparison of the fluorescence intensities of the different fluorochromes. Mean fluorescence intensities of *E. coli* cells after FISH using probe EUB338-I labeled with the respective fluorochrome are shown. Aside from the excitation and recording wavelengths, images were acquired using the same imaging settings to ensure comparability. Note the logarithmic scaling of the *y*-axis. At least 100 *E. coli* cells were evaluated for each fluorophore.

In this study, we developed a multicolor FISH approach that utilizes seven alternative fluorophores in addition to the established fluorescent dye FLUOS (Table 2). The alternative dyes employed here have already been used for other applications in the life sciences, such as the visualization of membrane proteins in human cell lines and chromosomes (e.g., Beliveau et al., 2015; Chamma et al., 2016). However, derivatives of these alternative dyes which can be bound to oligonucleotides became available only recently. The here presented approach is based on confocal microscopy with WLL technology that allows the precise adjustment of the excitation wavelengths and of the wavelength recording windows for emitted fluorescence. These features enable the concurrent use of fluorophores whose excitation and/or emission spectra show adjacent but still distinct maxima (Figure 1). To evaluate the suitability of these fluorophores for rRNA-targeted FISH, the *Bacteria*-specific probe EUB338-I (Amann et al., 1990) was separately mono-labeled with each dye and hybridized to cells from a log-phase *E. coli* culture. All fluorophores yielded clearly detectable signals that were suitable for the visualization of *E. coli* cells and allowed the quantification of fluorescence brightness by image analysis (Figure 2). Under the applied conditions, the alternative fluorophores Atto 565, Atto 594, and Atto 633 yielded strong fluorescence intensities per *E. coli* cell that were comparable to the canonical fluorophore Cy3 (Figure 2). These four dyes were considerably brighter than all other tested fluorophores, including the well-established dyes Cy5 and FLUOS (Figure 2). The dyes Atto 425 and Atto 490LS were relatively dim compared to the other fluorophores (Figure 2). However, proper adaptation of the laser power and detector settings permitted the acquisition of fluorescence images for each dye in the subsequent multicolor FISH experiments (see below). No fluorescent signals were observed in control hybridizations of *E. coli* cells to the nonsense probe NONEUB mono-labeled with each of the alternative fluorophores, confirming that none of these dyes attached non-specifically to the cells (data not shown).

The simultaneous application of different fluorophores in multicolor FISH experiments could be hampered by excitation crosstalk or emission bleed-through among the dyes, which would cause unspecific fluorescence signals. According to the spectral properties of the dyes used for multicolor FISH in this study (Figure 1), such problems are not expected if both the appropriate excitation wavelength and emission window is used to record the fluorescent signal of each dye. Consistently, in a multicolor FISH experiment using these dyes and mixed microbial pure cultures (mock community), no unspecific signals were observed (see below). However, the excitation spectrum of the canonical dye Cy3 suggests that excitation crosstalk likely occurs between Cy3 and the fluorophores Atto 532 or Atto 565, and the emission spectrum of Cy3 indicates that emission bleed-through occurs with Atto 532 and to a lesser extent with Atto 565 (Supplementary Figure S1). Similarly, both excitation crosstalk and emission bleed-through would be expected for a combination of the dyes Cy5 and Atto 633 (Supplementary Figure S1). These biases were experimentally confirmed (Supplementary Figure S2), so that Cy3 should not be combined with Atto 532 or Atto 565, and Cy5 not with Atto 633 in the same FISH experiment. In order to maximize the number of simultaneously applicable fluorophores, we excluded Cy3 from the subsequent multicolor FISH experiments. We also decided to use the brighter dye Atto 633 (Figure 2) instead of Cy5.

To assess whether the alternative fluorophores alter the melting behavior of the oligonucleotide probes, we recorded dissociation profiles for probe EUB338-I mono-labeled with each dye. Increasingly stringent hybridization and washing conditions (Daims et al., 2005) were applied in FISH experiments with the probes and *E. coli* as the target organism. The shape of the probe dissociation profiles was similar for the alternative fluorophores and the canonical dyes Cy3, Cy5, and FLUOS (Supplementary Figure S3). Merely with DY-681, the intensity of probe-conferred fluorescence started to decrease at a lower stringency than with the other dyes (Supplementary Figure S3). At high hybridization stringencies, this melting profile may lead to a slightly dimmer fluorescent signal of probes labeled with DY-681 compared to probes labeled with the other fluorochromes.

### Application of Alternative Fluorophores for Multicolor FISH

**FIGURE 3:**
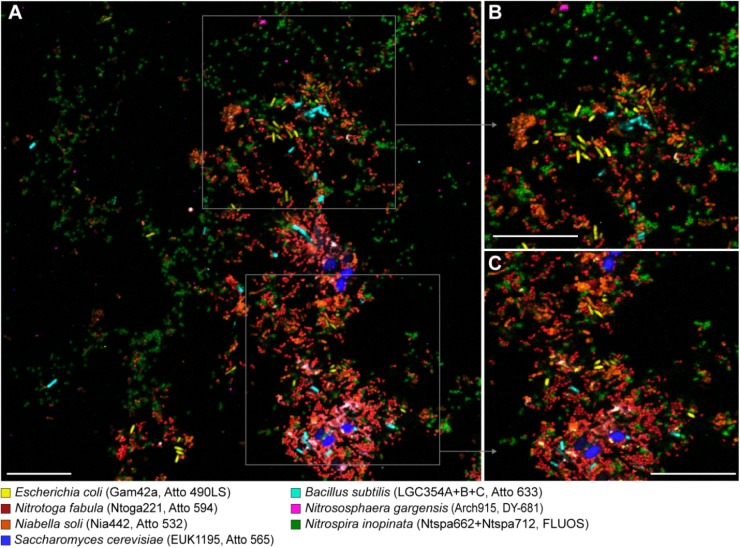
Simultaneous visualization, by multicolor FISH, of seven phylogenetically distinct microbial isolates in a mixture of pure cultures (mock community). The organisms, assigned false colors, rRNA-targeted oligonucleotide probes, and fluorochromes are indicated. **(A)** Representative image showing all organisms in the same microscopic field of view. Scale bar, 20 μm. **(B,C)** Enlarged parts of the image as indicated by the frames in panel **(A)**.

The use of the alternative fluorophores for multicolor FISH was demonstrated for a mixed mock community of seven microorganisms from different major phylogenetic groups: “*Candidatus* Nitrotoga fabula” (Betaproteobacteria), *E. coli* (Gammaproteobacteria), *B. subtilis* (Firmicutes), *Niabella soli* (Bacteroidetes), *Nitrospira inopinata* (Nitrospirae), *Nitrososphaera gargensis* (Thaumarchaeota), and *Saccharomyces cerevisiae* (Ascomycota). Oligonucleotide probes specifically targeting each of these organisms were labeled with different fluorophores (Table 1). Following FISH with these probes, all seven microorganisms in this mock community could be identified and clearly distinguished at the single cell level by confocal fluorescence microscopy (Figure 3). With the proper adjustments for the excitation wavelength and emission recording window for each dye (Table 2), no excitation crosstalk or emission bleed-through between any of the dyes was observed (Figure 3). Additional application of probe EUB338-I (labeled with Atto 425) resulted in the expected double-labeling of the bacterial cells with EUB338-I and the respective specific probe (Supplementary Figure S4). The use of Atto 425 also resulted in a weak fluorescent signal from the eukaryotic *S. cerevisiae* cells. A separate FISH experiment using a mixture of *E. coli* and *S. cerevisiae* cells, and probe EUB338-I labeled with dye Atto 633, revealed that *S. cerevisiae* was not erroneously detected by probe EUB338-I. Instead, the yeast cells showed autofluorescence with the imaging settings used to detect the signals of Atto 425 (Supplementary Figure S5).

**FIGURE 4:**
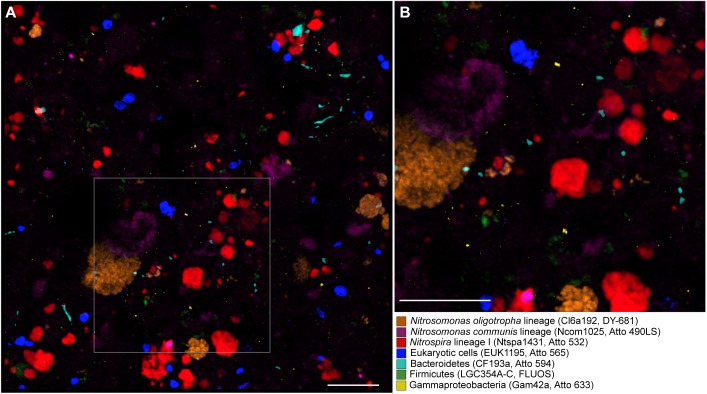
Simultaneous visualization, by multicolor FISH, of seven microbial target groups in activated sludge. The target organisms, assigned false colors, rRNA-targeted oligonucleotide probes, and fluorochromes are indicated. **(A)** Combined maximum intensity projection of confocal image stacks of each probe signal. Scale bar, 20 μm. The size of the image stacks along the *z*-axis was 12 μm. For the purpose of illustration, the brightness of the projection was enhanced by using the respective tool of the *daime* software. **(B)** Enlarged part of the image as indicated by the frame in panel **(A)**.

To test the applicability of multicolor FISH with the alternative fluorophores on a complex environmental sample, we concurrently visualized seven distinct microbial target groups in nitrifying activated sludge from a municipal WWTP in Klosterneuburg, Austria: two different lineages of betaproteobacterial AOB (*Nitrosomonas* cluster 6a and the *Nitrosomonas communis* cluster), NOB of *Nitrospira* lineage I, members of the Bacteroidetes, Firmicutes, and Gammaproteobacteria, and eukaryotes. Representatives of these phylogenetic groups are widespread in WWTPs (Juretschko, 2000; Daims et al., 2001; Adamczyk et al., 2003; Gruber-Dorninger et al., 2015; McIlroy et al., 2015; Foissner, 2016). All seven probe target groups were detected in the sludge flocs and could be distinguished from each other in the same multicolor FISH experiment (Figure 4). Control hybridizations were performed with the sludge and probe NONEUB mono-labeled with each of the alternative fluorophores. In these controls, no fluorescence was detected aside from background fluorescence of the sludge matrix, which was also observed after FISH with NONEUB labeled with the canonical dyes Cy3, Cy5, and FLUOS (data not shown).

Finally, the applicability of the fluorophores for sequential hybridization (i.e., subsequent hybridizations of the same sample at different formamide concentrations) was confirmed. For this purpose, a previously analyzed activated sludge sample from a municipal WWTP (Ingolstadt, Germany) was sequentially hybridized at three different formamide concentrations (50%, 35% and 25%) to three distinct rRNA-targeted oligonucleotide probes. These probes targeted NOB of *Nitrospira* lineage I, sublineage Ib, and sublineage Ig (Table 1; Gruber-Dorninger et al., 2015). Signals for all three targeted NOB groups were detected in the sludge flocs. Consistent with the previous analysis (Gruber-Dorninger et al., 2015), an overlay of the signals for lineage I (probe Ntspa1431) and sublineage Ib (probe Ntspa1131) was observed, whereas the signals for lineage Ig (probe Ntspa451) did not overlap with the signals of the other applied *Nitrospira* probes (Supplementary Figure S6).

### *In situ* Agarose Embedding for Multicolor FISH and Spatial Coaggregation Analyses of Nitrifiers

Prior to fixation for FISH, activated sludge from the WWTP Klosterneuburg was embedded in agarose and transferred onto microscope slides in order to reduce the impact of fixation and hybridization on the 3D structure of the microbial assemblies in the flocs. In particular, the agarose embedding avoided centrifugation of the biomass, which is part of the standard formaldehyde fixation protocol for rRNA-targeted FISH (Manz et al., 1992; Daims et al., 2005). This modified fixation procedure was fully compatible with multicolor FISH, as the applied agarose (LE Agarose, Biozyme) did not show autofluorescence that would hamper image acquisition (Figure 4).

In order to assess whether agarose embedding had an effect on the 3D structure of the sludge flocs, the spatial coaggregation patterns of nitrifying microorganisms in the flocs were quantified by image analysis. Previous studies had shown that AOB and NOB microcolonies often coaggregate in activated sludge. Biological reasons for this spatial arrangement are that the NOB feed on the nitrite produced by AOB, while the AOB benefit from the removal of toxic nitrite (Juretschko et al., 1998; Daims et al., 2006; Maixner et al., 2006). Consistently, our quantitative analysis confirmed a pronounced coaggregation of AOB and NOB over a distance range from ca. 0.5 to 12.5 μm in agarose-embedded sludge (Supplementary Figure S7). Beyond this range, the AOB and NOB microcolonies were randomly distributed (Supplementary Figure S7). Coaggregation was also detected for conventionally fixed sludge (i.e., fixed without agarose embedding but with centrifugation). However, the minimal coaggregation distance was slightly shorter (<0.5 μm) and random distribution started already at a distance of ca. 9 μm (Supplementary Figure S7). Hence, the spatial structure of the conventionally fixed sludge was slightly altered during fixation and hybridization. The quantified difference between the coaggregation patterns appears to be small. However, earlier analyses of a nitrifying biofilm suggested that steep *in situ* concentration gradients of nitrite exist within a short distance of 10 μm from AOB microcolonies. The nitrite concentration was highest close to the AOB, but it decreased rapidly and within a few μm distance because of diffusion and nitrite consumption by NOB (Maixner et al., 2006). Interestingly, different NOB populations colonized the biofilm along these nitrite gradients, apparently reflecting their different adaptations to elevated or low nitrite concentrations (Maixner et al., 2006). Thus, a high precision of coaggregation analyses is important for understanding interactions between tightly packed microorganisms that involve the production and consumption of diffusible compounds. The modified fixation protocol with embedding can improve the precision of spatial analyses based on FISH. Multicolor FISH offers the additional advantage that multiple microbial populations can be detected simultaneously (Figure 4), increasing the probability that new spatial arrangement patterns are found.

## Discussion

### Selection of Alternative Fluorophores and Use With Conventional Microscopes

In this study, we demonstrated a simple and efficient multicolor FISH approach. It is based on the use of alternative fluorophores in combination with WLL technology, which is becoming widely available as part of modern CLSM equipment. The approach enabled the simultaneous detection and visualization of seven distinct microbial taxa in the same hybridization experiment (or potentially eight taxa, if Atto 425 is used to label a specific probe instead of EUB338-I, see also Supplementary Figure S4B). In the analyzed samples, the fluorescence intensity of the alternative fluorophores was well sufficient for rRNA-targeted FISH of microbes with mono-labeled oligonucleotide probes. Most of the dyes showed a comparable or higher fluorescence intensity than the most commonly applied fluorophores Cy3, Cy5, and FLUOS (Figure 2). As with these canonical dyes, double-labeling (Stoecker et al., 2010) or multi-labeling (Schimak et al., 2015) of oligonucleotide probes with the same alternative fluorophore could further enhance the signal intensity, for example to detect organisms with a low ribosome content or to improve FISH results with autofluorescent samples. Because of excitation crosstalk and emission bleed-through, Cy3 and Cy5 were not compatible with Atto 532 and Atto 565 (Cy3) or Atto 633 (Cy5). This limitation should be considered if Cy3- or Cy5-labeled “legacy probes” must be used together with alternative fluorophores in multicolor FISH experiments. All other tested dyes can be freely combined, because unspecific signals due to crosstalk and bleed-through are unlikely based on the spectral properties of the fluorochromes (Figure 1) and were consistently not observed in any of our experiments.

The number of distinct target organisms, which can be distinguished by our multicolor FISH approach, is limited by the number of different fluorophores that can be detected by the imaging equipment and can be combined without excitation crosstalk or emission bleed-through. Here, up to eight fluorophores were applied together (Figure 1) and could be imaged using a CLSM equipped with a WLL and a UV diode. It should be possible to use subsets of the fluorophores (except DY-681) with more common CLSMs that are equipped with Ar and He/Ne lasers as well as an UV diode. Furthermore, the dyes Atto 425, Atto 565, and Atto 633 (as well as the canonical fluorophore FLUOS) can also be used with conventional epifluorescence microscopes and filter sets commonly used for imaging DAPI, Cy3, and Cy5 fluorescence signals. However, in these cases fewer microbial target groups can be visualized simultaneously.

### Comparison With Other Multicolor FISH Approaches

CLASI-FISH, which relies on the combinatorial labeling of microbial cells with multiple different fluorophores, can differentiate more than eight organisms in one experiment (Valm et al., 2011). However, the approach introduced here, uses a well-established standard protocol for FISH with rRNA-targeted DNA oligonucleotide probes, and does not depend on image post-processing or image analysis steps other than false-coloring of the obtained signals. In particular, our method, unlike all previously published multicolor FISH methods, does not rely on color blending. Thus, the imaging parameters can be freely adjusted to the signal intensity of each fluorophore and target organism, without affecting the correct identification of other organisms in the same experiment. Nevertheless, the alternative fluorophores can also be used for combinatorial labeling if phylogenetically nested probes are applied (Supplementary Figures S4B, S6), or in DOPE-FISH, (MiL)-FISH and CLASI-FISH protocols to further increase the number of organisms detectable by these methods. However, the same biases and constrains would apply to combinatorial labeling with the alternative fluorophores as for the conventional fluorophores used in previously published combinatorial labeling methods.

### Preservation of 3D Structures

Multicolor rRNA-targeted FISH is a powerful tool with many potential applications in microbial ecology and medical microbiology. For example, multicolor FISH can efficiently complement DNA sequencing-based microbiome studies by revealing the spatial arrangement of multiple different organisms in biofilms and other complex samples (Mark Welch et al., 2016). In this context, the preservation of 3D structures is important. Previous protocols improved the structure preservation during FISH by embedding already fixed samples in agarose, polyacrylamide, or polymerizing resin (Moter et al., 1998; Daims et al., 2001, 2006; Mark Welch et al., 2016). Alternatively, biofilm was fixed while still attached to its substratum and subsequently detached and cryosectioned for FISH analysis (Almstrand et al., 2013). The embedding of samples already prior to or during fixation, as applied in this study, can further improve structure preservation. However, the fixation and embedding protocol as applied in this study is only suitable for the FISH analysis of Gram-negative microorganisms, as formaldehyde fixation would hamper the FISH-based detection Gram-positive microorganisms (Braun-Howland et al., 1992). In addition, the embedding approach has only been tested with floccular samples (activated sludge) and will likely need adaptations for use with other types of samples such as sessile biofilms. As a next step, different embedding media (including those mentioned above) and fixation protocols could be tested to develop an optimized method for the pre-fixation embedding and subsequent multicolor FISH of flocs, biofilms, and tissue samples.

### Compatibility With Established Applications of FISH

The new multicolor FISH method is based on the standard FISH protocol, with the key modification that the rRNA-targeted oligonucleotide probes are labeled with non-canonical fluorophores. Experiments performed in this study have validated this method and showed that the selected fluorophores do not alter the hybridization behavior or specificity of rRNA-targeted oligonucleotide probes across a wide range of target organisms. Notably, the preparation of samples for this multicolor FISH approach does also not differ from standard FISH. Hence, we expect that in future studies no additional experimental controls beyond those applying to the standard FISH protocol need to be performed. In consequence, the multicolor FISH method should also be compatible with manual or automated quantitative FISH approaches that have been used in combination with the standard FISH protocol. These approaches, and their limitations, have been reviewed in detail elsewhere (Daims and Wagner, 2007). Approaches to physiologically characterize uncultured microorganisms, by combined single-cell isotope labeling, FISH, and chemical imaging (Wagner, 2009), provide both spatial and functional information about the target organisms. If combined with multicolor FISH, these tools could be used even more efficiently, for example to monitor the utilization of isotope-labeled substrates by multiple different organisms. Such applications should be facilitated by a straightforward multicolor FISH approach, which does not need complex image processing procedures and is easy to interpret. The method introduced here meets these requirements. It may become a valuable tool in future studies, which aim to verify “omics”-based hypotheses on the ecological roles of uncultured microorganisms by *in situ* functional tests at the single-cell level. In summary, our multicolor FISH method is straightforward, easy to implement, and should be compatible with virtually all applications of standard FISH with rRNA-targeted oligonucleotide probes.

## Author Contributions

PP, ML, and HD conceived the research idea and contributed to the development of the research plan. ML and MS performed the laboratory work. ML, PP, and HD carried out image analysis and evaluated the data. ML, PP, and HD were the primary authors writing the manuscript, while all other authors were involved in writing and editing of the manuscript.

## Conflict of Interest Statement

The authors declare that all research was conducted in the absence of commercial or financial relationships that could be construed as a conflict of interest.

## Supplementary Material

FIGURE S1 Excitation and emission spectra of the fluorochromes used for multicolor FISH and of Cy3 and Cy5. **(A)** Excitation spectra. Wavelengths indicated after the fluorochrome names and by vertical lines are the excitation wavelengths applied for imaging. For Atto 425 and DY-681, different wavelengths than the maxima of the excitation spectra were used because of technical limitations of the microscopy equipment. **(B)** Emission spectra. Shaded regions indicate the wavelength windows used for recording the fluorescence emitted by the respective dyes. The spectral properties of the fluorochromes are listed in Table 2 in the main text. RU, relative units.

FIGURE S2 Excitation crosstalk and emission bleed-through of the dyes Cy3 and Cy5 with alternative fluorophores used in this study. Mean fluorescence intensities of *Escherichia coli* cells after FISH using probe EUB338-I labeled with the respective fluorochrome are shown. “Imaging settings” refers to the applied excitation wavelength and emission recording window. Note the logarithmic scaling of the *y*-axes. At least 100 *E. coli* cells were evaluated for each combination of fluorophore and imaging settings. RU, relative units. **(A)** Analysis of the fluorophores Atto 532, Cy3, and Atto 565. **(B)** Analysis of the fluorophores Atto 633 and Cy5.

FIGURE S3 Dissociation profiles of probe EUB338-I labeled with the different fluorochromes. *E. coli* was used as the target organism in FISH experiments with increasingly stringent hybridization and washing conditions. Data points depict the mean fluorescence intensity of at least 100 *E. coli* cells at each formamide concentration in the hybridization buffer. Error bars (s.e.m.) are not shown if smaller than symbols. Probe dissociation curves (dashed lines) were approximated by non-linear regression with a sigmoidal regression model. Fluorochromes are indicated above the plots. Note the different scaling of the *y*-axes. RU, relative units.

FIGURE S4 Simultaneous visualization, by multicolor FISH, of seven phylogenetically distinct microbial isolates in a mixture of pure cultures (mock community). The organisms, assigned false colors, rRNA-targeted oligonucleotide probes, and fluorochromes are indicated. **(A)** Representative image showing all organisms in the same microscopic field of view. Scale bar, 20 μm. **(B)** Same field of view as in panel **(A)**, but showing the fluorescence signal of probes EUB338-I to III targeting most bacteria. Several rod-shaped *B. subtilis* cells, which appear in panel **(B)**, could not be visualized in panel **(A)**. The binding sites of probes EUB338 and LGC354 at the 16S rRNA overlap, so that a more efficient hybridization of one probe can lead to a very dark fluorescent signal of the respective other probe. Scale bar, 20 μm.

FIGURE S5 Visualization of a mixture of *E. coli* and *S. cerevisiae* cells. *E. coli* was stained by FISH with the EUB338 probe mix labeled with Atto 633 (false-colored in red). S. cerevisiae was not labeled by FISH. Instead, the autofluorescence of the yeast cells (false-colored in cyan) was recorded using the same imaging settings as would be needed for recording the signals of dye Atto 425. Scale bar, 10 μm.

FIGURE S6 Simultaneous visualization of three *Nitrospira* target groups after sequential FISH at three different formamide concentrations. The applied probes were labeled with alternative fluorochromes. The target organisms, assigned false colors, rRNA-targeted oligonucleotide probes, and fluorochromes are indicated. Scale bar, 20 μm.

FIGURE S7 Spatial arrangement pattern analysis of ammonia- and nitrite-oxidizing bacteria in activated sludge. The sludge was either embedded in agarose prior to formaldehyde fixation or conventionally fixed in formaldehyde without embedding. The horizontal dashed line (*y* = 1) indicates random spatial distribution at the respective distance. Values above this line indicate coaggregation (i.e., NOB cells were more abundant at this distance from AOB cells than expected for randomly distributed populations). Values below this line indicate that NOB cells were less abundant at this distance from AOB cells than expected for randomly distributed populations. Data points are means from the analysis of *n* = 23 confocal *z*-stacks. Shaded areas depict 95% confidence intervals of the mean. RU, relative units.
