# Supplementary figures and images for: A Multicolor Fluorescence in situ Hybridization Approach Using an Extended Set of Fluorophores to Visualize Microorganisms
Source: Front Microbiol. 2019 Jun 19;10:1383. doi: 10.3389/fmicb.2019.01383 (PMC6593226; doi:10.3389/fmicb.2019.01383)

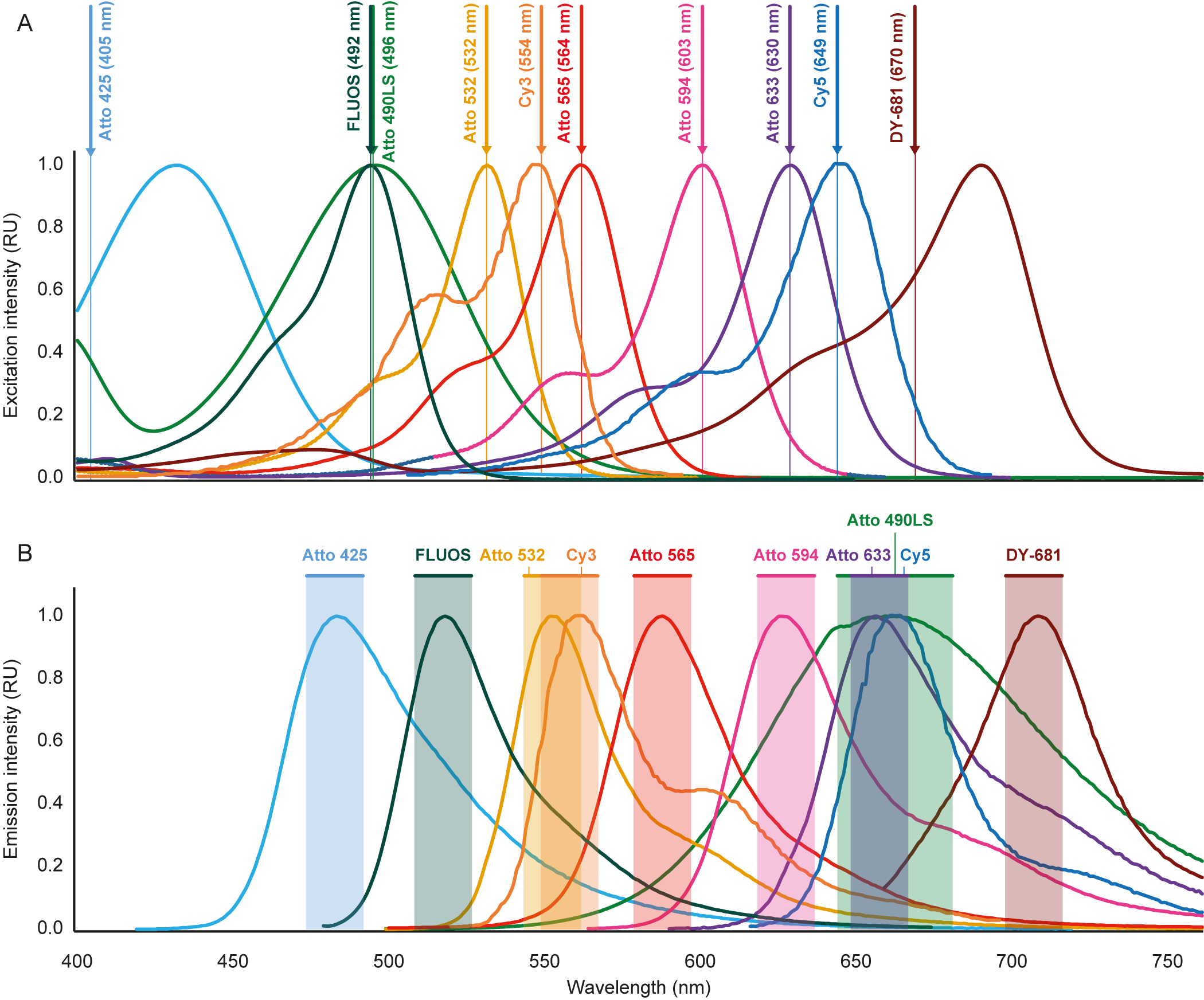

Supplement: FIGURE S1 — Excitation and emission spectra of the fluorochromes used for multicolor FISH and of Cy3 and Cy5. (A) Excitation spectra. Wavelengths indicated after the fluorochrome names and by vertical lines are the excitation wavelengths applied for imaging. For Atto 425 and DY-681, different wavelengths than the maxima of the excitation spectra were used because of technical limitations of the microscopy equipment. (B) Emission spectra. Shaded regions indicate the wavelength windows used for recording the fluorescence emitted by the respective dyes. The spectral properties of the fluorochromes are listed in Table 2 in the main text. RU, relative units. [file Image_1.TIF]

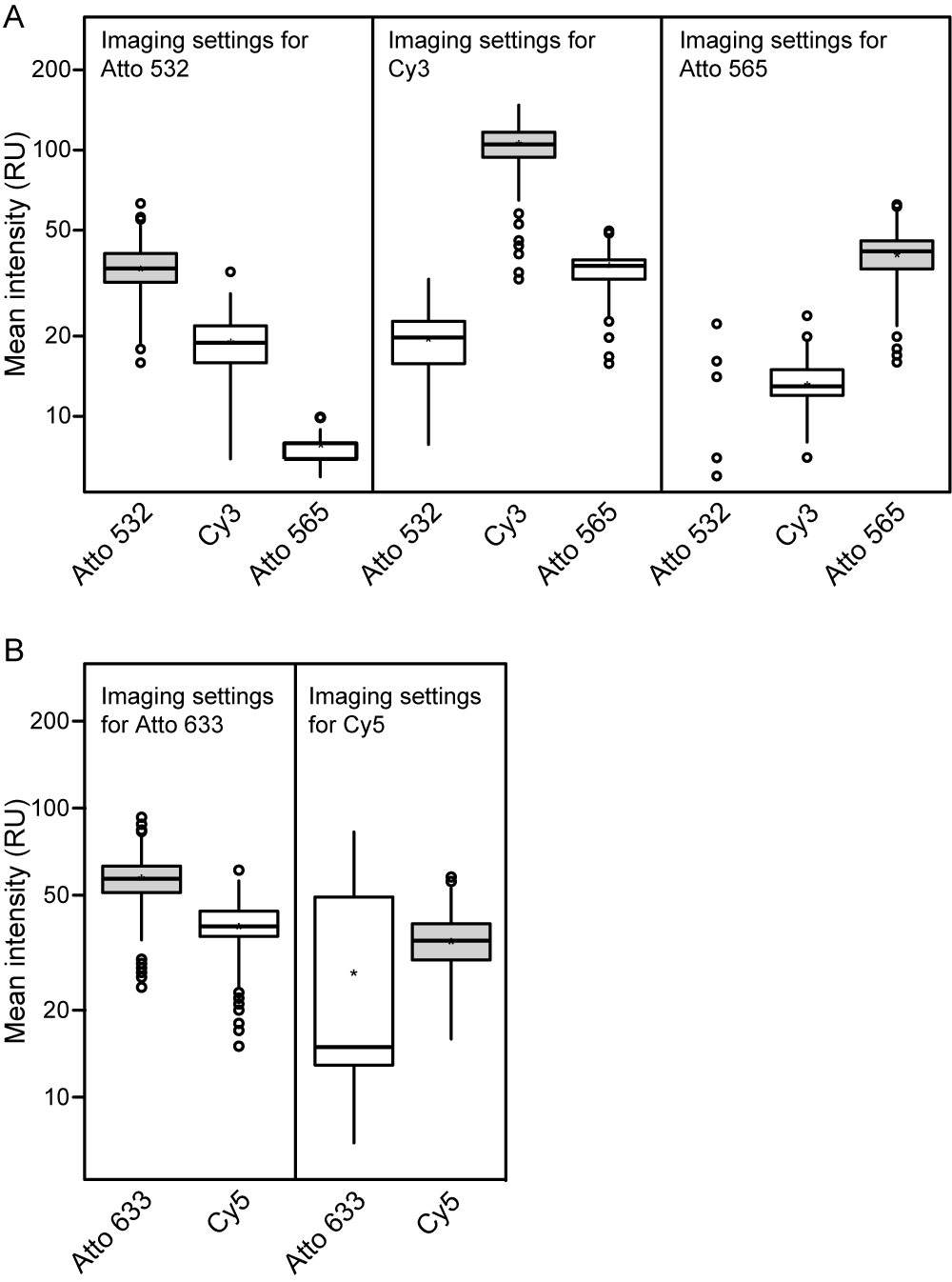

Supplement: FIGURE S2 — Excitation crosstalk and emission bleed-through of the dyes Cy3 and Cy5 with alternative fluorophores used in this study. Mean fluorescence intensities of Escherichia coli cells after FISH using probe EUB338-I labeled with the respective fluorochrome are shown. “Imaging settings” refers to the applied excitation wavelength and emission recording window. Note the logarithmic scaling of the y-axes. At least 100 E. coli cells were evaluated for each combination of fluorophore and imaging settings. RU, relative units. (A) Analysis of the fluorophores Atto 532, Cy3, and Atto 565. (B) Analysis of the fluorophores Atto 633 and Cy5. [file Image_2.TIF]

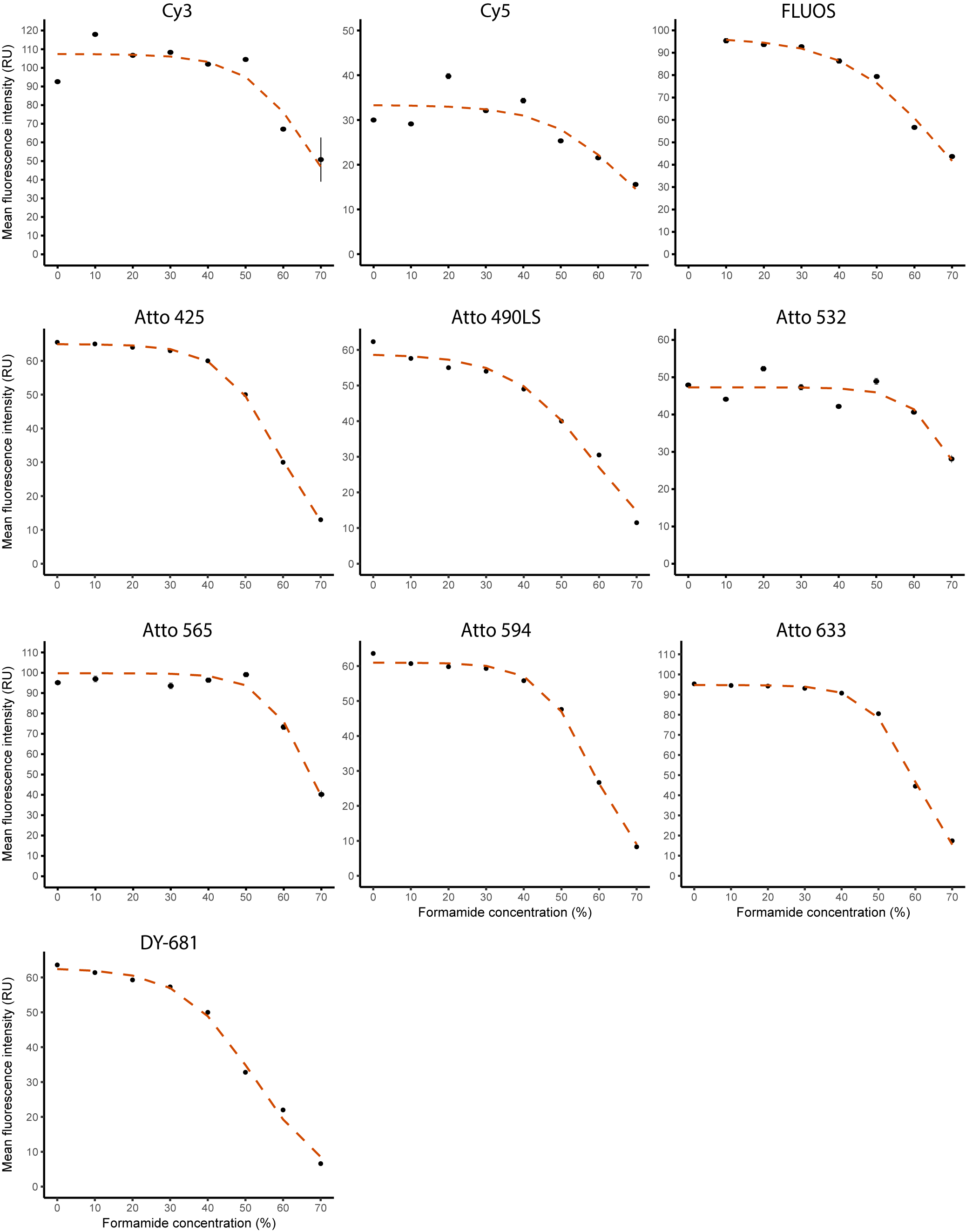

Supplement: FIGURE S3 — Dissociation profiles of probe EUB338-I labeled with the different fluorochromes. E. coli was used as the target organism in FISH experiments with increasingly stringent hybridization and washing conditions. Data points depict the mean fluorescence intensity of at least 100 E. coli cells at each formamide concentration in the hybridization buffer. Error bars (s.e.m.) are not shown if smaller than symbols. Probe dissociation curves (dashed lines) were approximated by non-linear regression with a sigmoidal regression model. Fluorochromes are indicated above the plots. Note the different scaling of the y-axes. RU, relative units. [file Image_3.TIF]

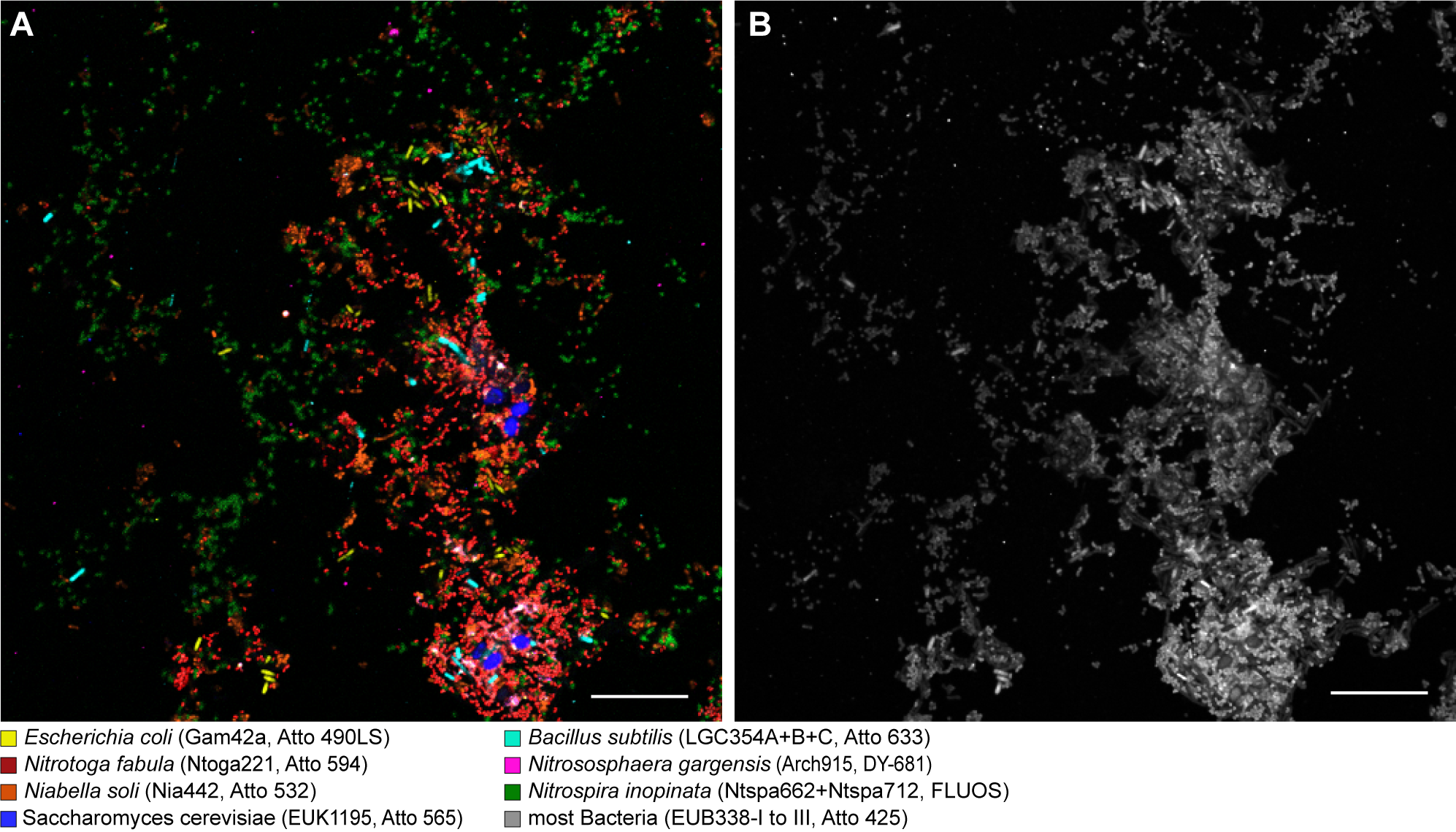

Supplement: FIGURE S4 — Simultaneous visualization, by multicolor FISH, of seven phylogenetically distinct microbial isolates in a mixture of pure cultures (mock community). The organisms, assigned false colors, rRNA-targeted oligonucleotide probes, and fluorochromes are indicated. (A) Representative image showing all organisms in the same microscopic field of view. Scale bar, 20 μm. (B) Same field of view as in panel (A), but showing the fluorescence signal of probes EUB338-I to III targeting most bacteria. Several rod-shaped B. subtilis cells, which appear in panel (B), could not be visualized in panel (A). The binding sites of probes EUB338 and LGC354 at the 16S rRNA overlap, so that a more efficient hybridization of one probe can lead to a very dark fluorescent signal of the respective other probe. Scale bar, 20 μm. [file Image_4.TIF]

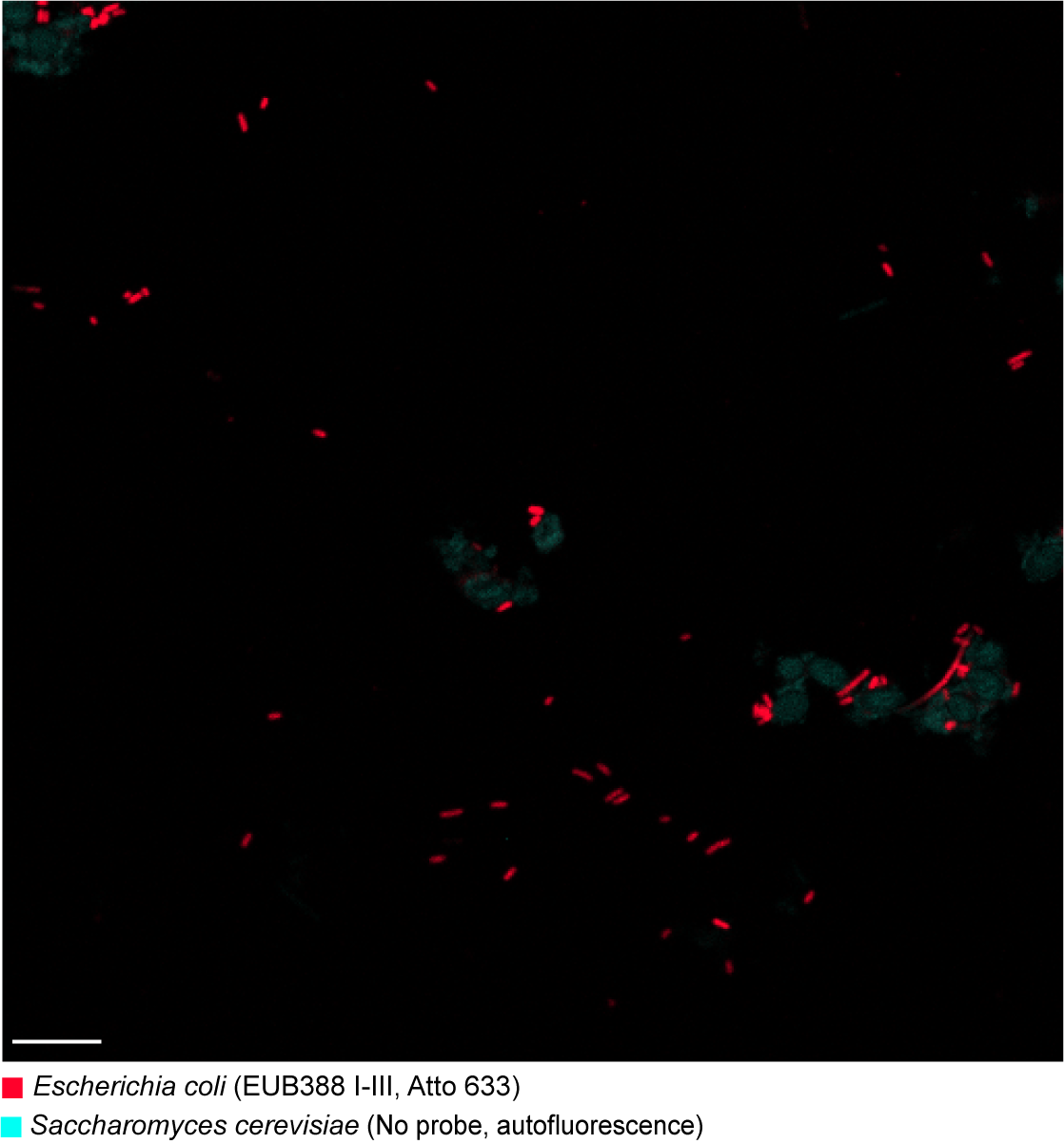

Supplement: FIGURE S5 — Visualization of a mixture of E. coli and S. cerevisiae cells. E. coli was stained by FISH with the EUB338 probe mix labeled with Atto 633 (false-colored in red). S. cerevisiae was not labeled by FISH. Instead, the autofluorescence of the yeast cells (false-colored in cyan) was recorded using the same imaging settings as would be needed for recording the signals of dye Atto 425. Scale bar, 10 μm. [file Image_5.TIF]

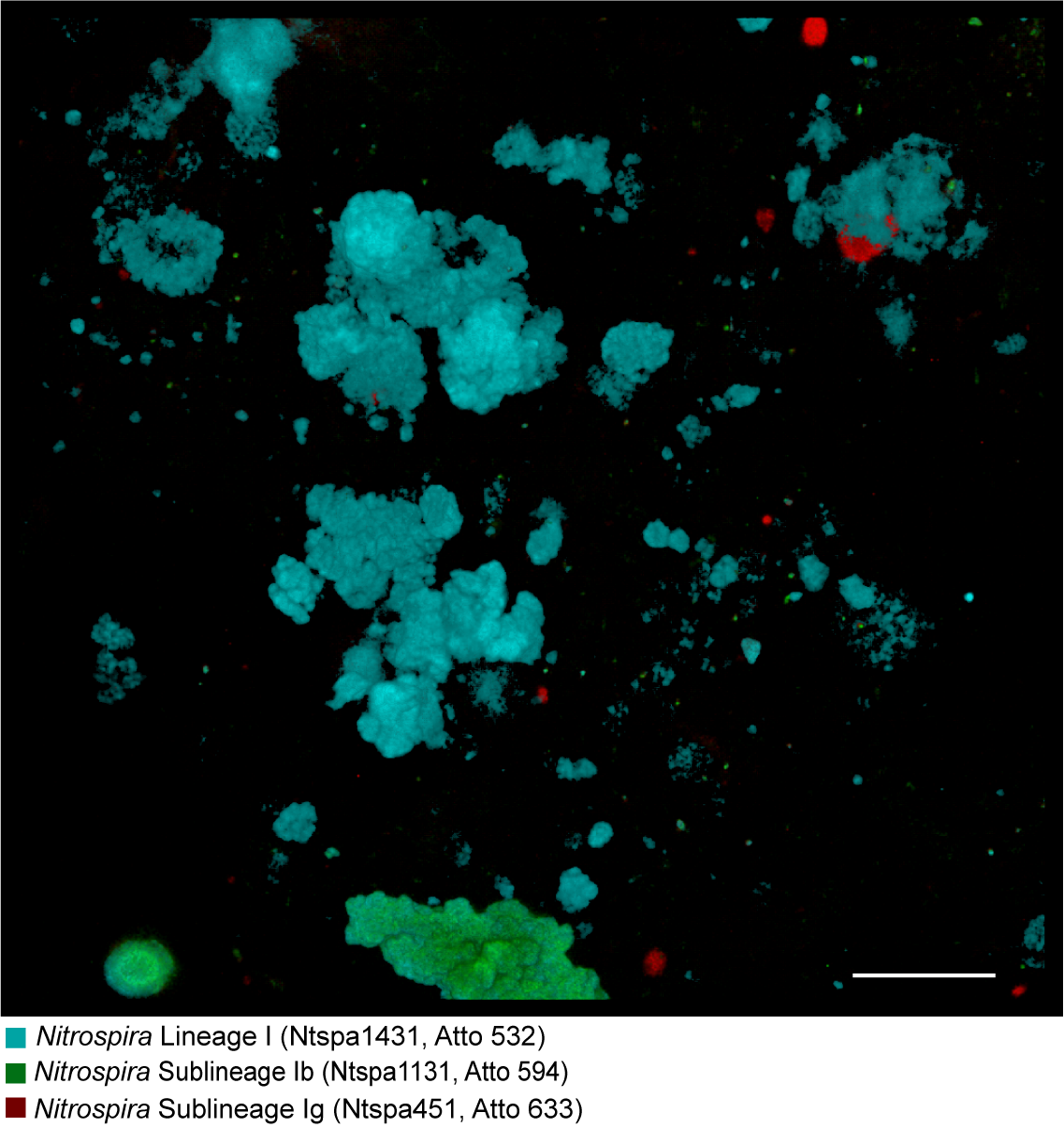

Supplement: FIGURE S6 — Simultaneous visualization of three Nitrospira target groups after sequential FISH at three different formamide concentrations. The applied probes were labeled with alternative fluorochromes. The target organisms, assigned false colors, rRNA-targeted oligonucleotide probes, and fluorochromes are indicated. Scale bar, 20 μm. [file Image_6.TIF]

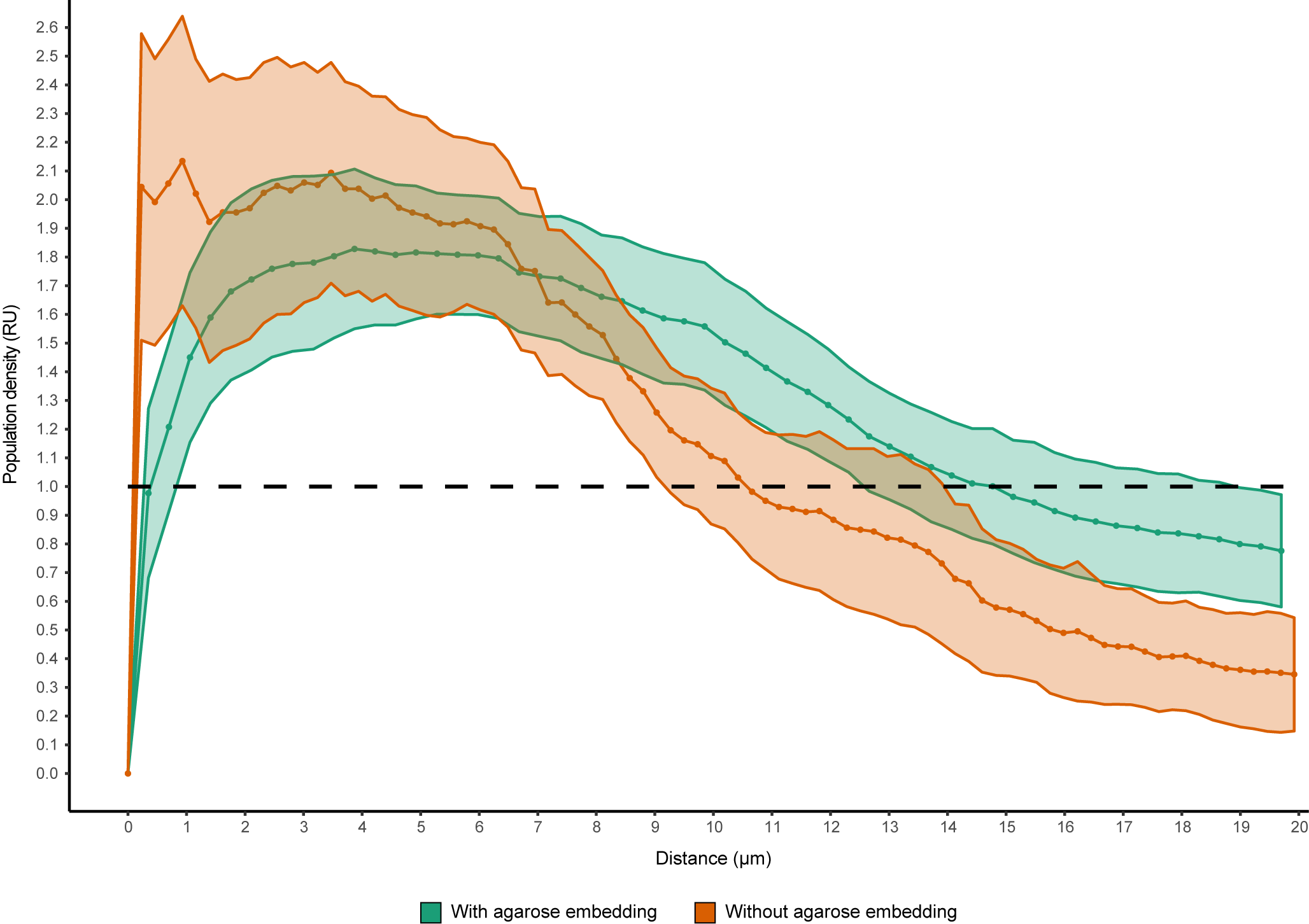

Supplement: FIGURE S7 — Spatial arrangement pattern analysis of ammonia- and nitrite-oxidizing bacteria in activated sludge. The sludge was either embedded in agarose prior to formaldehyde fixation or conventionally fixed in formaldehyde without embedding. The horizontal dashed line (y = 1) indicates random spatial distribution at the respective distance. Values above this line indicate coaggregation (i.e., NOB cells were more abundant at this distance from AOB cells than expected for randomly distributed populations). Values below this line indicate that NOB cells were less abundant at this distance from AOB cells than expected for randomly distributed populations. Data points are means from the analysis of n = 23 confocal z-stacks. Shaded areas depict 95% confidence intervals of the mean. RU, relative units. [file Image_7.TIF]
